# A scoping review of clinical *Āyurgenomics* studies incorporating *Prakṛti* (somatic constitution) assessment and genetic analysis and discussion of their relevance in primary healthcare

**DOI:** 10.3389/fmed.2026.1894441

**Published:** 2026-08-12

**Authors:** Aravind Kumar, Paul Morehead, Sangeeta Sharma, Supaya Wenuganen

**Affiliations:** 1 Department of Śālākya Tantra (Āyurvedic Ophthalmology and ENT), Amrita School of Ayurveda, Amrita Vishwa Vidyapeetham, Kollam, Keralam, India; 2 Department of Physiology and Health, College of Integrative Health and Medicine, Maharishi International University, Fairfield, IA, United States; 3 Department of Regenerative Organic Aggriculture and Center for Brain, Cognition, and Consciousness, Maharishi International University, Fairfield, IA, United States

**Keywords:** *Āyurgenomics*, case control, cross sectional, genetics, *Prakṛti*

## Abstract

**Background:**

*Āyurgenomics*, the integration of *Prakṛti* (somatic constitution) with genetics, seeks to rigorously analyze fundamental concepts of *Āyurveda* while incorporating this knowledge into modern practice. It has enormous scope for integration into the P4 systems of medicine (predictive, preventive, personalized, and participatory) by predicting disease susceptibility, exploring disease profiles, and planning therapeutic targets. This scoping review aims to explore published studies on *Āyurgenomics* that incorporate both *Prakṛti* analysis and genetic assessment.

**Methods:**

The scoping review was conducted using the Joanna Briggs Institute’s methodology for scoping reviews and reported using the Preferred Reporting Items for Systematic Reviews and Meta-Analyses Extension for Scoping Reviews (PRISMA-ScR) guidelines. Searches were conducted using the term “ayurgenomics” both individually and in combination with other terms in PubMed, Scopus, ScienceDirect, and Semantic Scholar. The studies were grouped into three broad categories based on their area of coverage.

**Results:**

16 clinical studies meeting the inclusion criteria out of a total of 281 were included for analysis. The studies were exclusively from India and published from the years 2012 to 2026. Out of the 16 studies, 13 were cross-sectional, while the remaining 3 were case–control. The total sample size was 3,695 subjects. Four methods of assessing *Prakṛti*, and a diverse range of genetic analyses were employed across all studies. Most of the studies (*n* = 13) could not be interpreted based on already-existing data as they are first-of-their-kind analyses. The relevance and application of these studies and *Āyurgenomics* as a whole in the primary healthcare sector may be understood under applicable parameters such as early-risk identification, personalized counseling, strengthening public health, low-cost risk stratification, and integration of traditional medicine with modern healthcare.

**Conclusion:**

This scoping review analyzes research on *Āyurgenomics* that incorporates both *Prakṛti* analysis and genetic assessment, and discusses their relevance in the primary healthcare sector. Further robust studies with larger samples may be employed to add to and enhance already-existing knowledge about this novel area of exploration in *Āyurveda*.

## 1 Introduction

*Āyurveda* is a natural, holistic system of health care that originated in ancient India (1, 2). It is formed from two words, *viz*., *Āyus* (life) and *Veda* (knowledge), thus, translating to the “science or knowledge of life.” (3) It is classified under the banner of Traditional, Complementary, and Alternative Medicine (TCAM) by the National Institutes of Health (NIH). It offers a comprehensive understanding of life, health, longevity, and therapeutic aspects (4). Its use in India is still quite prominent, with a growing global interest (4, 5). A study determined that *Āyurveda*, with its “natural approach” and “fewer side effects,” was preferred for managing non-communicable diseases (NCDs) by patients in the Organization for Economic Cooperation and Development (OCED) (4, 6).

A core tenet of *Āyurveda* is the principle of *Tridoṣās* or three somatic humors, *viz*., *Vāta*, *Pitta*, and *Kapha* (7, 8). These govern the physiological functions of the body at both microscopic and macroscopic levels by their own characteristics and functions (2). *Vāta* aids in transfer and movement within the body, transmission of molecules, nerve impulses, manifestation of shape, cell division, signaling, elimination of waste, thought, and normalizing the function of the other two humors (2, 9, 10). *Pitta* regulates various metabolic processes, such as digestion, metabolism, energy balance, thermoregulation, vision, pigmentation, and host surveillance (2, 11). *Kapha* oversees and governs structure and cohesiveness, storage, stability, maintenance, and development (2).

*Prakṛti* or somatic constitution originates under the influence of increased *Doṣās* at conception brought about by dietary and lifestyle habits. It is a set of physical, physiological, and psychological characteristics that is believed to be unchanged throughout the lifespan (8, 12–14). It is a vital clinical denominator that influences diagnosis and course of disease and dictates the prescription of pharmacological, dietary, and lifestyle protocols (15–17).

The classification of *Prakṛti* into various types aligns with scientific understanding of differentiation of individuals according to genetic, epigenetic, metabolic, and other factors (4). The relative predominance of *Doṣās* forms the basis for the sevenfold classification of *Prakṛti*, *viz*., three extreme types based on individual *Doṣās* (*Vātaja*, *Pittaja*, and *Kaphaja*), three dual types based on the combination of two *Doṣās1* (*Vāta-Pittaja*, *Vāta-Kaphaja*, and *Pitta-Kaphaja*), and one balanced type (*Vāta-Pitta-Kapha*) (14). The dual classification is further subclassified into six by contemporary scholars (*Vāta-Pitta*, *Pitta-Vāta*, *Vāta-Kapha*, *Kapha-Vāta*, *Pitta-Kapha*, *and Kapha-Pitta*) for a total of ten types of *Prakṛti* (14, 18–20).

*Prakṛti* allows a trait-based assessment based upon its conceptual foundation in the *Tridoṣā* theory, observable traits and features, and independence from *Vikṛti* or pathological transient states (4). Its appropriation into approximately 200–250 characteristics based on various physical, physiological, and psychological factors, which indicate the multidimensional construct of observable features and latent traits as seen in *Prakṛti* (4, 21).

Although a causal relationship between *Prakṛti* and modern genetics was hypothesized years ago, there exists no mutual contradiction between the two, and as such, reasons explained in modern genetics may explain the existence of *Prakṛti*. Although both *Tridoṣās* and *Prakṛti* encapsulate foundations of personalized precepts in *Āyurveda* for implementation in diagnosis and therapy, it is crucial to ascertain and create their molecular basis (2, 22).

The intersection of *Āyurveda* and genomics may be complementary and supportive to the P4 systems of medicine, and, by extension, it may benefit primary healthcare (2, 23, 24). Personalized medicine, which evaluates a client based on the state of health, diet, lifestyle, and age, employs a diverse range of therapeutic methods to manage the same ailments in different individuals. Recent advances in personalized medicine enable prediction of disease susceptibility and early detection through profiling at the genetic, genomic, and individual levels (4). The intricate interplay between genetic, epigenetic, and environmental changes in DNA sequencing contributes to multiple phenotypic variants. Genetic expression within and among different populations provides insight into the molecular basis of phenotypic diversity and patterns of expression in disease.

*Āyurgenomics,* the integration of *Prakṛti* with modern genetics, holds promise for further exploration into personalized medicine and can function as a framework for understanding tenets of personalized therapy (2, 25). It rigorously evaluates and enables the understanding of basic concepts while incorporating practical approaches of *Āyurveda* into modern genetics (3). It performs the following: identification of molecular signatures that correlate with an individual’s *Prakṛti;* utilization of *Prakṛti* to stratify genetic studies; exploration of disease predisposition using traditional techniques of phenotyping; and development of personalized therapeutic strategies based on *Prakṛti* and genomic profiles (25). Its clinical applications encompass stratifying burden and risk of disease, planning of personalized dietary and lifestyle targets, drug development and pharmacogenomics, and application in integrative research for global health requirements (25). Its challenges include standardization of *Prakṛti* assessment among different practitioners, identification of genetic heterogeneity among diverse global populations, bridging epistemological gaps between *Āyurveda* and conventional biomedicine, and identification of ethical and regulatory concerns (25).

Research conducted on *Āyurgenomics* encompasses literature reviews and clinical studies. Review articles analyzed conceptual foundations and assessment of *Prakṛti* with several physiological and clinical parameters. Clinical studies on *Āyurgenomics* include cross-sectional and case–control studies. These evaluate conditions by either associating them with *Prakṛti* alone or a combination of *Prakṛti* analysis and genetic assessment. The aim of this scoping review is to collect all available clinical studies of *Āyurgenomics* that evaluate both *Prakṛti* and genetic assessment, analyze their results, and recommend potential areas of future research in this relatively novel spectrum of *Āyurvedic* exploration.

## 2 Methods

### 2.1 Reporting methodology

This scoping review was conducted according to the Joanna Briggs Institute’s (JBI) methodology for scoping reviews (26, 27) and is reported according to the Preferred Reporting Items for Systematic Reviews Extension for Scoping Reviews (PRISMA-ScR) guidelines (26, 28, 29).

### 2.2 Eligibility criteria

Sources were considered eligible for inclusion if they assessed both *Prakṛti* and genetics and recruited individuals of any age, gender, and origin. Clinical research on human subjects, such as case–control studies, cross-sectional studies, and other pertinent clinical studies, were also considered. Studies reporting *Prakṛti* analysis and genetic assessment were considered eligible regardless of sample size and parameter assessed.

Review articles, book chapters, animal studies, experimental studies, and duplicate publications were excluded from this review. Clinical studies assessing either *Prakṛti* or genetics individually were also excluded.

Year of publication was not accorded an absolute timeframe of selection due to the limited and emerging nature of research in this field. Incorporating diverse study designs was deemed appropriate due to the available evidence amounting to only select types of study. The inclusion and exclusion criteria for this study are described in Table 1.

**Table 1:** Eligibility criteria for studies.

Parameter	Inclusion criteria	Exclusion criteria
Population	Human participants of any age, gender, or origin	Molecular or animal studies
Context	Clinical studies on *Āyurgenomics* incorporating *Prakṛti* assessment and genetic analysis	Studies assessing either *Prakṛti* or genetics only
Study design	Case–control, cross-sectional, observational, clinical studies	Review articles, book chapters, experimental studies, articles lacking sufficient data, conceptual studies
Outcomes	Association between *Prakṛti* and specific condition/parameter	Studies with insufficient outcome-related data
Language	Articles published in the English language	Articles published in a language other than English
Publication period	No restriction	—
Publication status	Full-text articles available to the authors	Articles not available to authors and duplicate publications

### 2.3 Sources of information

Literature searches were conducted on multiple electronic databases such as PubMed, Scopus, ScienceDirect, and Semantic Scholar. Additional literature searches were manually screened from reference lists of relevant journal publications.

### 2.4 Search strategy

A search strategy was initiated by incorporating keywords with Boolean operators. The term “ayurgenomics” was searched both individually and with other terms including “prakriti,” “Ayurvedic constitution,” “personalized medicine,” “duchenne muscular dystrophy,” “obesity,” “type 2 diabetes,” “rheumatoid arthritis,” and “gut microbiome.” Boolean operators were incorporated between “ayurgenomics” and the subsequent terms. Table 2 describes the search strategy for each database.

**Table 2:** Search terms.

Database	Search terms/keywords used	Date searched
PubMed	(“ayurgenomics”) AND (“prakriti” OR “Ayurvedic constitution” OR “personalized medicine” OR “duchenne muscular dystrophy” OR “obesity” OR “type 2 diabetes” OR “rheumatoid arthritis” OR “gut microbiome”)	April–May 2026
Scopus	Search strategy using “ayurgenomics” and keywords related to Prakrti, personalized medicine, and diseases	April–May 2026
ScienceDirect	Search strategy using “ayurgenomics” and keywords related to Prakrti, personalized medicine, and diseases	April–May 2026
Semantic scholar	Search strategy using “ayurgenomics” and keywords related to Prakrti, personalized medicine, and diseases	May 2026

### 2.5 Data extraction, charting, validation, and variables

Data extraction, charting, and validation were done in accordance with the objectives of this scoping review. A pre-prepared Microsoft Excel spreadsheet was used to extract and chart the data. Extraction, charting, and verification were conducted by two independent reviewers (PM and SW). Any disparities, disagreements, or confusion regarding the extracted data were resolved by consensual discussion between the first two reviewers with the aid of a third reviewer (SS).

The selected studies’ data were analyzed under the attributes of study participants, assessment of Prakrti, and genetic analysis. The following variables were extracted from the studies to facilitate mapping and synthesis of the literature: (1) author; (2) year of publication; (3) sample size; (4) type of clinical study; (5) method of *Prakṛti* assessment; (6) method of genetic analysis; and (7) findings.

### 2.6 Application of the Strengthening the Reporting of Genetic Association Studies (STREGA) checklist

The STREGA checklist, an extension of the Strengthening the Reporting of Observational Studies in Epidemiology (STROBE) checklist, assesses 22 parameters under the following heads: title and abstract (1 item), introduction (2 items), methods (9 items), results (5 items), discussion (4 items), and other information (1 item). Each item is identified by indicating the page number or numbers of the article where it is located (30).

### 2.7 Qualitative assessment

Quality assessment assessed risk of bias and was executed using the Appraisal Tool for Cross-Sectional Studies (AXIS) (31) and the Newcastle-Ottawa scale for case–control studies (32). The AXIS tool assesses 20 criteria under the following heads: introduction (1 criterion), methods (10 criteria), results (5 criteria), discussion (2 criteria), and other (2 criteria). Answers to each criterion are either Yes, No, or Do not Know/Comment (31). The Newcastle–Ottawa Scale for Case–Control Studies allocates asterisks based upon the responses to the following criteria: selection (1 star), comparability (up to 2 stars), and exposure (1 star each). Each criterion has one or more questions under it, to which stars are allocated. Selection has four questions, comparability has one, and exposure has three. Higher scores on the scale indicate lower risks of bias (32).

### 2.8 Synthesis of results

Narrative analysis and tabulation were done to map the characteristics and scope of the studies on *Āyurgenomics* incorporating both *Prakṛti* assessment and genetic analysis. The studies were summarized based on their characteristics, tools for *Prakṛti* assessment, technique of genetic analysis, and results obtained. The studies were grouped based on the conditions and physiological parameters assessed.

Quantitative synthesis or meta-analysis was not considered for this review due to heterogeneity of study designs, evolving conceptual frameworks, and a limited amount of high-quality clinical evidence. Emphasis was placed on the association between *Prakṛti* and genetics that resulted in key observations for vulnerability toward disease and physiological expression.

## 3 Results

### 3.1 Study selection

**Figure 1:**
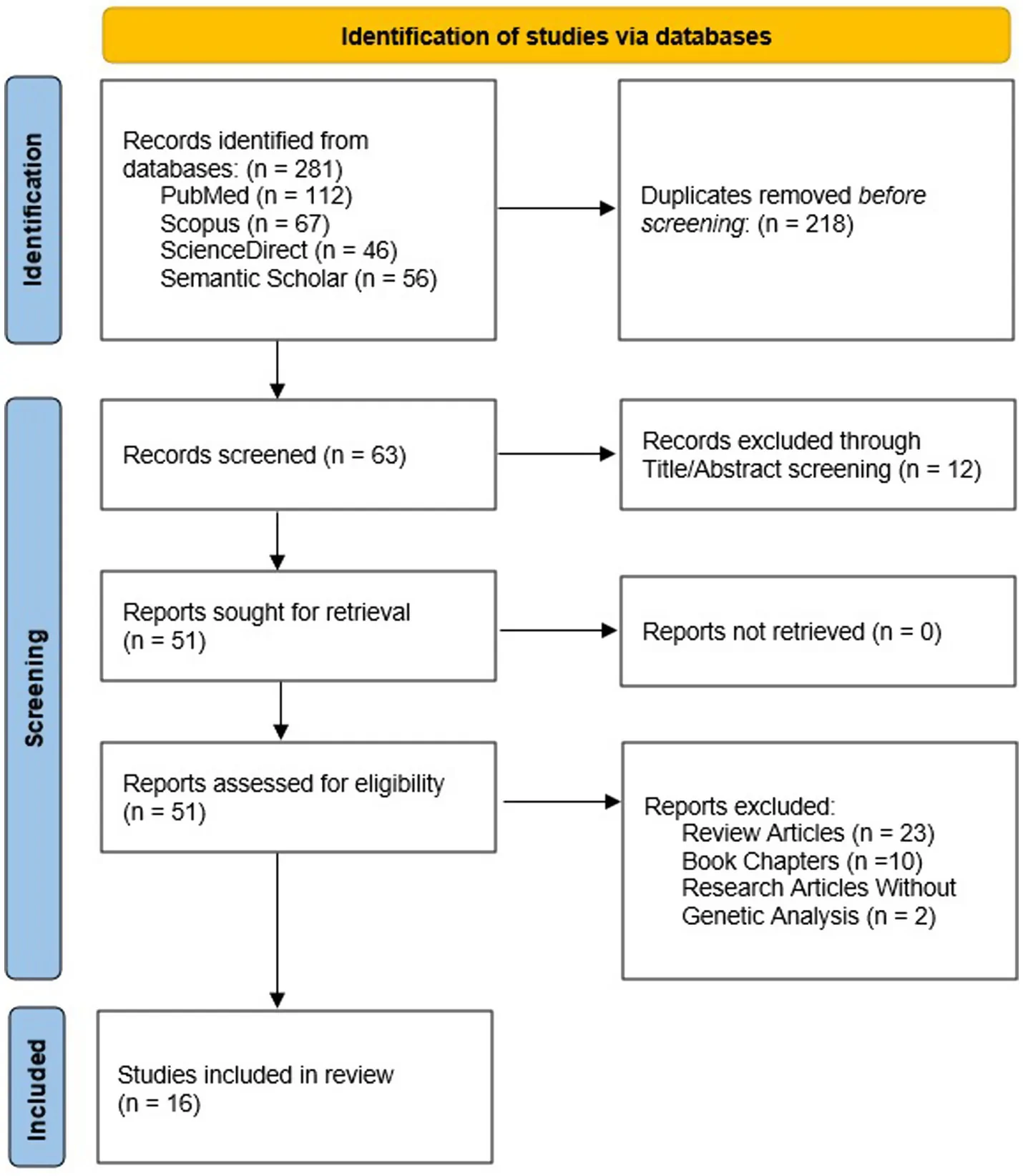
PRISMA workflow diagram. The PRISMA diagram was obtained from https://www.prisma-statement.org/prisma-2020-flow-diagram. The Diagram was adapted based on the methodology of the article.

A total of 281 articles were identified, out of which 218 were excluded due to being duplicates. 63 articles underwent primary screening, out of which 12 were excluded due to filtering by Title/Abstract. A total of 51 reports were retrieved and assessed for eligibility, out of which 23 review articles, 10 book chapters, and 2 clinical studies that incorporated only *Prakṛti* assessment were excluded. A total of 16 clinical studies incorporating the assessment of both *Prakṛti* and genetics were included in this review. The workflow of record identification for this study is demonstrated in a PRISMA flow diagram. (Figure 1).

### 3.2 Types of studies

Three articles (33–35) were case–control studies, while 13 articles (36–48) were cross-sectional studies.

### 3.3 Sample size

**Figure 2:**
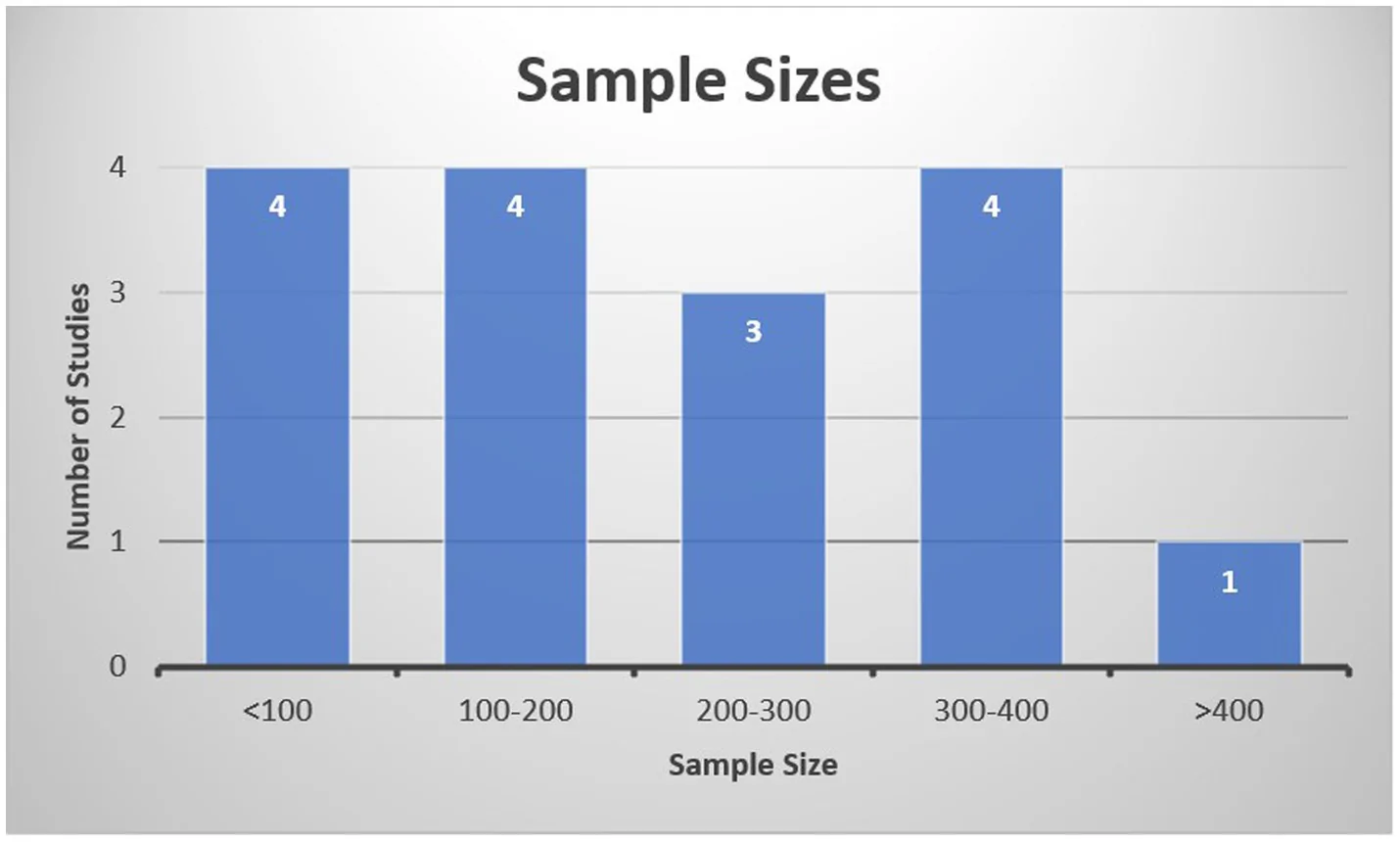
Sample sizes for the studies.

A total of 3,695 subjects were recruited across all studies. Four studies (41, 43, 45, 48) recruited less than 100 subjects; four (33, 35, 37, 39) recruited between 100 and 200 subjects; three (40, 46, 47) recruited between 200 and 300 subjects; four (34, 36, 38, 44) recruited between 300 and 400 subjects; and one (42) recruited more than 400 subjects. (Figure 2).

### 3.4 Prakṛti assessment

**Figure 3:**
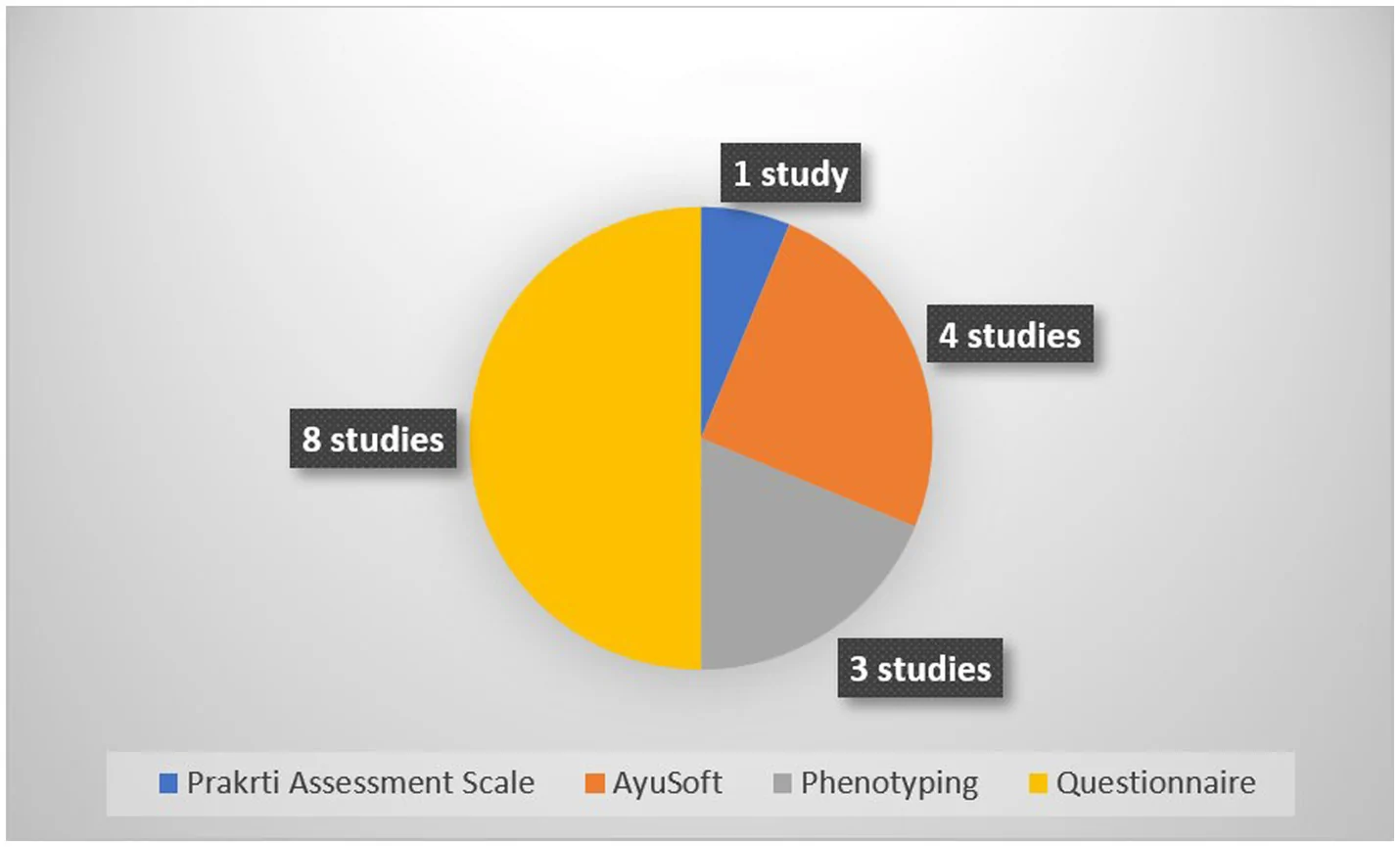
*Prakṛti* assessment tools.

Four types of *Prakṛti* analysis tools were employed across all studies. One study employed the *Prakrti* Assessment Scale developed by the Central Council for Research in Ayurvedic Sciences (CCRAS) (36). AyuSoft, a decision-support system developed by the Center for Development in Advanced Computing, Pune, was utilized by four studies (37, 46–48). Three studies assessed *Prakrti* by phenotyping (39, 41, 45), while eight studies employed validated questionnaires (33–35, 38, 40, 42–44). (Figure 3).

### 3.5 Genetic analysis

Genetic analyses included different types of polymerase chain reaction (PCR) (33, 35–38, 42); multiplex ligation-dependent probe amplification (MLPA) (37); next-generation sequencing (NGS) (37); DNA extraction and analysis (34, 38); Sanger sequencing and allele mining (33); whole exome sequencing and variant calling (39); genotyping and genome-wide association studies (40); lymphoblastoid cell line generation, lymphoblastoid cell line (LCL) generation, LCL growth curve assay, and carboxyfluorescein succinimydil ester (CFSE) proliferation assay (41); 16S rRNA gene amplification sequencing (42); analysis of single-nucleotide polymorphisms (SNPs) (43, 45); isolation of both DNA and RNA, genotyping and validation of DNA, and cDNA microarray experiments (44); metagenomic sequencing (46); high-throughput genotyping and sequencing (47); and DNA methylation (48). The characteristics of all studies assessed in this review are described in Table 3.

**Table 3:** Characteristics of studies included in the scoping review.

Authors	Sample size	*Prakṛti* analysis	Genetic analysis	Other testing
Saini et al., 2026 (36)	300	CCRAS *Prakṛti* Assessment Scale	Quantitative polymerase chain reaction (QPCR)	Olfactory performance using the *Sniffin Sticks* test
Trivedi et al., 2026 (37)	120	AyuSoft	Multiplex polymerase chain reaction (MPCR), multiplex ligation-dependent probe amplification (MLPA), next-generation sequencing (NGS)	Biochemical analysis of CPK
Murugan et al., 2025 (38)	300	Questionnaire	Polymerase chain reaction-restriction fragment length polymorphism (PCR-RFLP)	—
Singh et al., 2024 (33)	112	Questionnaire	Genomic DNA extraction, amplification by polymerase chain reaction, Sanger sequencing, and allele mining	—
Abbas et al., 2022 (39)	108	Phenotypic assessment using traditional criteria	Whole exome sequencing and variant calling	Mapping and enrichment analysis, multisystem phenotype association, replication analysis
Juyal et al., 2022 (40)	244	Validated questionnaire	Genotyping, genome-wide association study	—
Juyal et al., 2012 (34)	350 cases, 376 controls	Questionnaire	DNA extraction and analysis	Height, weight, BMI, blood pressure, swelling, blood/serum examination, X-rays, MRI scanning; Hb%, ESR, RA factor, anti CCP
Chakraborty et al., 2021 (41)	10	Detailed clinical phenotyping	Lymphoblastoid cell line generation, LCL growth curve assay, CFSE proliferation assay	—
Chauhan et al., 2018 (42)	528	Questionnaire	16S rRNA gene amplification sequencing, qPCR validation	—
Aggarwal, et al., 2015 (43)	96	Questionnaire	Analysis of single nucleotide polymorphisms from the IGVC panel Phase II	—
Prasher et al., 2008 (44)	320	Questionnaire	DNA isolation, genotyping, validation, RNA isolation, cDNA microarray experiments	33 biochemical markers (hematology and biochemistry)
Aggarwal et al., 2010 (45)	96	Phenotyping	Analysis of single nucleotide polymorphisms	—
Shalini et al., 2021 (46)	272	AyuSoft	Metagenomic sequencing	—
Govindaraj et al., 2015 (47)	262	AyuSoft	High throughput genotyping and resequencing	—
Gupta et al., 2018 (35)	54	Questionnaire	Polymerase chain reaction and restriction digestion	—
Rotti et al., 2015 (48)	147	AyuSoft	Methylated DNA immunoprecipitation and microarray analysis	—

### 3.6 Pertinent findings

The parameters of both *Prakṛti* assessment and genetic analysis were used in the grouping of the studies. A total of three groups were identified: (1) disease, (2) parameters of health, and (3) miscellaneous. Parameters evaluated in the disease group included muscular dystrophies (37), obesity (38), type II diabetes mellitus (33, 35), hypoxia (43), and rheumatoid arthritis (34, 40). Olfactory function (36), gut and oral microbiome (42, 46), and high-altitude adaptation (45) were evaluated under the parameters of health group. In the miscellaneous group, biochemical parameters (44), risk for disease (39), specific genetic analysis (41), genome-wide analysis (47), and DNA methylation (48) were assessed. Pertinent findings from all studies are described in Table 4.

**Table 4:** Pertinent findings from the studies.

Study	Parameter	Findings
Disease		
Trivedi et al., 2026 (37)	Muscular dystrophies	*Kaphapradhāna Pittānubandha*, *Kaphapradhāna Vātānubandha*, and *Vātapradhāna Pittānubandha* are the dominant *Prakṛti* groups in patients with Duchenne muscular dystrophy (DMD) and Becker muscular dystrophy (BMD). *Kaphapradhāna Pittānubandha Prakṛti* was 46.67% in both DMD and BMD groups. DMD had 40.95% *Kaphapradhāna Vātānubandha Prakṛti* and 12.38% *Vātapradhāna Kaphānubandha Prakrti*. BMD had 53.33% *Kaphapradhāna Vātānubandha Prakṛti*, while *Vātapradhāna Kaphānubandha Prakrti* was absent. DMD patients exhibited more deletions, duplications, and point variants, while BMD exhibited more deletions. Variants detected included frameshift deletions, noncoding single-nucleotide variants (SNV), frameshift insertions, and nonsense SNV.
Murugan et al., 2025 (38)	Obesity	70 subjects under *Vāta*, 67 under *Pitta*, and 13 under *Kapha* were in the normal group. 30 subjects under *Vāta*, 33 under *Pitta*, and 87 under *Kapha* were in the obese group. Prevalence of the risk allele was 50% in the obese group as compared to 38% in the normal group. The wild-type genotype (AA) was present in 40 individuals with the *Vāta* type, 28 with the *Pitta* type, and 22 with the *Kapha* type. The heterozygous type of genotype (AG) was present in 51 individuals with the *Vāta* type, 49 with the *Pitta* type, and 44 with the *Kapha* type. The mutant type of genotype (GG) was present in 9 individuals with the *Vāta* type, 23 with the *Pitta* type, and 22 with the *Kapha* type. Statistically significant association between *Kapha Prakṛti* and obesity has been determined.
Singh et al., 2024 (33)	Type II diabetes mellitus	*Kapha* and *Pitta Prakṛti* individuals were diagnosed with type 2 diabetes Changes due to both synonymous and non-synonymous mutations were shown to contribute to the manifestation of type II diabetes mellitus.
Gupta et al., 2018 (35)		*Kaphaja* and *Kapha-Pitta Prakṛti* were strong risk factors for type II diabetes mellitus A significant association between the MTHFR-C677T single-nucleotide polymorphism (SNP) and type II DM was observed. No association between *Prakṛti* and the A1298 variant of the MTHFR gene was identified.
Juyal et al., 2022 (40)	Rheumatoid arthritis	*Prakṛti-*specific novel disease risk genes of high effect sizes were identified. SNPs identified in the *Vāta* cohort did not associate with the *Pitta* and *Kapha* cohorts. Three genes identified in the *Kapha* cohort were not found in either *Vāta* or *Pitta* cohorts.
Juyal et al., 2012 (34)		Inflammatory genes are determinants in the *Vāta* subgroup, while oxidative stress pathway genes are observed in *Pitta* and *Kapha* groups. Characteristics such as severity were more pronounced in the *Vāta* subgroup. *PON2*, *IL1,β*, and a trend of association of *TNF α* were observed in the *Vāta* subgroup. *SOD3* and a trend of *PON1* were observed in the *Pitta* subgroup. No significant association with any markers was observed in the *Kapha* subgroup
Aggarwal, et al., 2015 (43)	Hypoxia	Significant differences in allele frequencies were observed in seven genes after the false discovery rate (FDR) correction. A non-synonymous variation associated with thrombosis/bleeding susceptibility differed significantly between *Kapha* and *Pitta* constitution types. A combination of *EGLN1* and ancestral *VWF* allele was significantly higher in *Kapha* than in *Pitta*.
Health parameters		
Saini et al., 2026 (36)	Olfactory function	Hyposmia was found to be more in *Vātika Prakṛti* and less in *Kapha Prakṛti*. Normosmia was found to be more in *Kapha Prakṛti* and less in *Vātika Prakṛti*. Supersmeller was found in *Pitta Prakṛti*. Downregulation of OR6K3 gene was observed in *Pitta* and *Kapha Prakṛti*. OR10Z1 was upregulated in *Vātika Prakṛti* OR6K3 has maximum expression in *Vātika Prakṛti* followed by *Kapha* and *Pitta*, while OR10Z1 has maximum expression in *Kapha Prakṛti* followed by *Pitta* and *Vāta Prakṛti*.
Chauhan et al., 2018 (42)	Gut and oral microbiome	Exclusion of Bacteriodetes and Firmicutes showed lesser diversity and richness among females with *Pitta* but higher evenness than *Vāta* and *Kapha* subgroups. Male samples showed similarity in diversity, richness, and evenness across all *Prakṛti* groups. Over-presentation of seven species of gut microbiota.
Shalini et al., 2021 (46)		Pathogenic organisms in the gut microbiome were found to be higher in *Kaha Prakṛti* than in *Pitta*. Pathogenic organisms in the oral microbiome were found to be higher in *Vāta Prakṛti* and least abundant in *Pitta Prakṛti*. *Paraprevotella* and Christensenellaceae was observed in *Vāta* individuals.
Aggarwal et al., 2010 (45)	High-altitude adaptation	A link between high-altitude adaptation and *EGLN1* was observed. TT genotypes, which are more frequent in *Kapha* types, were associated with patients suffering from pulmonary edema, but is lower in *Pitta* individuals and absent in high-altitude natives Disparate genetic lineages at high altitudes share the same ancestral allele that is over-represented in *Pitta* and positively associated with higher altitudes.
Miscellaneous		
Prasher et al., 2008 (44)	Biochemical parameters	Lipid profiles were higher in *Kapha* than in *Pitta* and *Vāta*, except HDL. Uric acid was also elevated in *Kapha*. Prolactin and prothrombin were elevated in *Vāta*, while hemoglobin, PCV, and RBC counts varied significantly in all three groups. Differential expression of genes among *Prakṛti* types was enriched in core biological processes. Significant enrichment of housekeeping, disease-related, and hub genes were observed in the constitution types.
Abbas et al., 2022 (39)	Risk for disease	Activation of a natural killer cell involved in immune response and the tricarboxylic acid were found to be enriched in *Vāta-Pitta* and *Pitta-Kapha* groups. Aldehyde biosynthetic process and neuron migration were enriched in *Vāta-Kapha* and *Pitta-Kapha* cohorts. Novel SNPs were identified that could explain trajectories for immune diseases, bleeding, or thrombosis.
Chakraborty et al., 2021 (41)	Specific genetic analysis	Significant differences in cell proliferation rates were observed such that *Kapha* proliferates significantly slower than *Vāta* and *Pitta*. *Vāta* shows higher cell death in response to ultraviolet (UV), but shows an inherently higher rate of proliferation. CDK2 phosphorylation differed in the levels of *Vāta<Pitta<Kapha*, while mitosis marker histone 3 phosphorylation differed in the levels of *Kapha<Vāta<Pitta*.
Govindaraj et al., 2015 (47)	Genome-wide analysis	52 SNPs were identified as genuine characteristics of *Prakṛti;* further exploration revealed distinct characteristics in different *Prakṛti* groups. Categorization with an unknown *Prakṛti* model showed that 7 individuals belonged to *Vāta*, 20 to *Pitta*, and 11 to *Kapha* in a sample of 297 Indians. Four markers of *PGM1* were associated with *Pitta* and not the other *Doṣās*.
Rotti et al., 2015 (48)	DNA methylation	A total of 23,101, 23,276, and 21,104 significantly methylated probes were observed in *Kapha*, *Pitta*, and *Vāta Prakṛti*, respectively. A higher distribution of probes was associated with the gene body in *Pitta Prakṛti* as opposed to *Kapha Prakṛti*. *Pitta Prakṛti* phenotypes, characterized by higher metabolism, showed distinct promoter and gene body methylation. *Vāta Prakṛti*, characterized by motion, showed DNA methylation in 52 promoters and 139 CpG islands. *Kapha Prakṛti* showed DNA methylation in 23 promoters and 19 CpG islands, respectively.

### 3.7 Analysis with the STREGA checklist

Seven studies (33, 38, 39, 42, 44, 47, 48) identified and reported potential sources of bias and their management (Option 9). Two studies (38, 42) had specific sections on outcome data (Option 15). Three studies (34, 43, 44) assessed parameters other than *Prakṛti* analysis and genetic assessment (Option 17). Twelve studies (33–35, 37, 38, 41–47) discussed the generalizability and variability of the study results (Option 21). Eleven studies (33–42, 46) declared sources of funding or the role of specific funders for which the study was based (Option 22) (Table 5).

**Table 5:** Strengthening the reporting of genetic association studies (STREGA) checklist.

Item	Page numbers in the article															
	Saini et al., 2026 (36)	Trivedi et al., 2026 (37)	Murugan et al., 2025 (38)	Singh et al., 2024 (33)	Abbas et al., 2022 (39)	Juyal et al., 2022 (40)	Juyal et al., 2012 (34)	Chakraborty et al., 2021 (41)	Chauhan et al., 2018 (42)	Aggarwal, et al., 2015 (43)	Prasher et al., 2008 (44)	Aggarwal et al., 2010 (45)	Shalini et al., 2021 (46)	Govindaraj et al., 2015 (47)	Gupta et al., 2018 (35)	Rotti et al., 2015 (48)
1	1	1	1	494	1	1	1	903	1	1	1	18,961	1	1	146	1
2	2	2	2	494–495	2	1–2	1–2	903–904	1–2	2	2	18,961	1–2	1–2	146–147	2
3	2	2	3	494	2	1	2	904	2	2	2	18,961	2	2	147	2
4	2	2	3	495	3	2	2	904	2	2	2	18,965	2	2, 8	147	2
5	2	2	3	495	3	2	2	903	2	2	2	18,965	2	2, 8	147	2
6	3	3	3	495	3	2	2	904	2	2	2	18,965	2	2, 8	147	2
7	3	2–3	3–4	495	3–4	3	2–3	904–905	2–3	2–3	3–4	18,961–18,963	2–4	2, 8	147	2–3
8	3	2–3	3	495	3–4	3	2–3	904–905	3	3	3–4	18,961	3–4	2, 8	147	2–3
9	—	—	10	495	5	—	—	—	8	—	5	—	—	2, 8–9	—	—
10	3	3	3	495	3	2	3	904	2	2	3	18,961	2	2	147	—
11	3	2	4	495–496	4–5	3	3	905	2–3	2	4–5	18,964	3–4	2–3, 8–9	147	2–3
12	3	3	4	496	4	3	3	906	4	3	4–5	18,966	—	10, 11	147	3
13	3	3	3	495	3	2	2	904	2	3	2	18,964	2	2, 8	147–148	2
14	3	4–6	4	495	4	3	2	904	2–3	2	4–5	18,963–18,964	2–4	2, 8	147–148	2
15	—	—	4	—	—	—	—	—	4	—	—	—	—	—	147–148	5–7
16	3–4	3–7	4	496	5–11	3	3	906	4–6	4	5–8	18,961	5	2	148	5–7
17	—	—	—	—	—	—	2	—	—	3	4–5	—	—	—	—	—
18	3–4	3–7	4	496	5–11	3	3	906	4–6	4–8	5–8	18,961	5–7	2	148	8–10
19	5	8	10	500	14	6	5–7	911	—	9	—	—	—	—	148	9
20	5	8–10	11	497	12–14	5–7	5–7	909–911	7–9	8–9	8–10	18,964–18,965	7–14	2–3, 7	148–149	8–10
21	—	9	11	497	—	—	5–7	909–911	9	9	8–10	18,964–18,965	7–14	2–3, 7	149	10
22	6	10	11	500	15	8	1, 7	912	10	—	—	—	15	—	149	—

### 3.8 Qualitative results

Qualitative assessment was conducted to understand the nature of the evidence, identify strengths and limitations, and to better understand heterogeneous data in the studies. The AXIS tool and Newcastle-Ottawa Scale were used to qualitatively analyze the studies.

### 3.9 Appraisal Tool for Cross-Sectional Studies

As the 13 cross-sectional studies (33–48) did not have non-responders and risk and outcome variables, options 7 to 9 were not applicable to them. Option 13 was answered as “No” across all studies due to the absence of non-responder bias. The absence of funding sources and conflicts of interest across all studies made the answer to Option 19 “No” across the board. Responses to the other criteria were “Yes” for all studies (Table 6).

**Table 6:** Appraisal tool for cross-sectional studies (AXIS).

Parameter	Criteria No.	Saini et al., 2026 (36)	Trivedi et al., 2026 (37)	Murugan et al., 2025 (38)	Abbas et al., 2022 (39)	Juyal et al., 2022 (40)	Chakraborty et al., 2021 (41)	Chauhan et al., 2018 (42)	Aggarwal, et al., 2015 (43)	Prasher et al., 2008 (44)	Aggarwal et al., 2010 (45)	Shalini et al., 2021 (46)	Govindaraj et al., 2015 (47)	Rotti et al., 2015 (48)
Introduction	1	Yes	Yes	Yes	Yes	Yes	Yes	Yes	Yes	Yes	Yes	Yes	Yes	Yes
Methods	2	Yes	Yes	Yes	Yes	Yes	Yes	Yes	Yes	Yes	Yes	Yes	Yes	Yes
	3	Yes	Yes	Yes	Yes	Yes	Yes	Yes	Yes	Yes	Yes	Yes	Yes	Yes
	4	Yes	Yes	Yes	Yes	Yes	Yes	Yes	Yes	Yes	Yes	Yes	Yes	Yes
	5	Yes	Yes	Yes	Yes	Yes	Yes	Yes	Yes	Yes	Yes	Yes	Yes	Yes
	6	Yes	Yes	Yes	Yes	Yes	Yes	Yes	Yes	Yes	Yes	Yes	Yes	Yes
	7	N/A	N/A	N/A	N/A	N/A	N/A	N/A	N/A	N/A	N/A	N/A	N/A	N/A
	8	N/A	N/A	N/A	N/A	N/A	N/A	N/A	N/A	N/A	N/A	N/A	N/A	N/A
	9	N/A	N/A	N/A	N/A	N/A	N/A	N/A	N/A	N/A	N/A	N/A	N/A	N/A
	10	Yes	Yes	Yes	Yes	Yes	Yes	Yes	Yes	Yes	Yes	No	Yes	Yes
	11	Yes	Yes	Yes	Yes	Yes	Yes	Yes	Yes	Yes	Yes	Yes	Yes	Yes
Results	12	Yes	Yes	Yes	Yes	Yes	Yes	Yes	Yes	Yes	Yes	Yes	Yes	Yes
	13	No	No	No	No	No	No	No	No	No	No	No	No	No
	14	N/A	N/A	N/A	N/A	N/A	N/A	N/A	N/A	N/A	N/A	N/A	N/A	N/A
	15	Yes	Yes	Yes	Yes	Yes	Yes	Yes	Yes	Yes	Yes	Yes	Yes	Yes
	16	Yes	Yes	Yes	Yes	Yes	Yes	Yes	Yes	Yes	Yes	Yes	Yes	Yes
Discussion	17	Yes	Yes	Yes	Yes	Yes	Yes	Yes	Yes	Yes	Yes	Yes	Yes	Yes
	18	Yes	Yes	Yes	Yes	Yes	Yes	Yes	Yes	Yes	No	Yes	Yes	Yes
Other	19	No	No	No	No	No	No	No	No	No	No	No	No	No
	20	Yes	Yes	Yes	Yes	Yes	Yes	Yes	Yes	Yes	Yes	Yes	Yes	Yes

### 3.10 Newcastle–Ottawa scale for case control studies

Controls were not explicitly defined in one study (34), and hence, no score could be accorded under that criterion. All other criteria were met, with the total score of one study (34) as 8 and the others (33, 35) as 9 (Table 7).

**Table 7:** Newcastle–Ottawa scale for case–control studies.

Study	Item and Score								Total score
	Is the case definition accurate? (1)	Representativeness of the cases (1)	Selection of Controls (1)	Definition of controls (1)	Comparability of cases and controls on the basis of the design or analysis (2)	Ascertainment of exposure (1)	Same method of ascertainment for cases and controls (1)	Non-response rate (1)	
Juyal et al., 2012 (34)	1	1	1	—	2	1	1	1	8
Singh et al., 2024 (33)	1	1	1	1	2	1	1	1	9
Gupta et al., 2018 (35)	1	1	1	1	2	1	1	1	9

## 4 Discussion

Sixteen studies incorporating both *Prakṛti* analysis and genetic assessment were included in this scoping review. All studies were conducted in India. Different methods of *Prakṛti* analysis and genetic assessment were used by the studies, with each one having its own benefits. The scope of information derived from the studies resulted in their categorization into disease (musculoskeletal disorders, obesity type II diabetes mellitus, and rheumatoid arthritis), parameters of health (olfactory function, gut and oral microbiome, and high-altitude adaptation), and miscellaneous (biochemical investigation, whole-genome analysis, and risk for disease).

Interpretation based on previously published data could not be done for all studies as they were the first studies of their kind. The results obtained in the study on obesity (38) are in line with the fundamental fact that the dominance of *Kapha Doṣa* has a greater affinity for obesity due to its propensity for gaining weight (49). The results obtained in the studies evaluating type II diabetes mellitus (33, 35) are partly in line with the results obtained by Mahalle et al. (50),in which *Kapha* is the dominant *Doṣa* among all three studies. However, the main difference between Mahalle et al. and the other two studies is that one of the *Prakṛti* assessed was predominant in *Vāta* and *Kapha* (50, 51).

The overall methodological quality across the studies was moderate-to-high. Analysis using the STREGA checklist indicated adequate reporting of study designs, participant characteristics, laboratory methods, and statistical analyses. The 13 cross-sectional studies fulfilled the majority of the criteria in the AXIS criteria, with clear study objectives, appropriate methods, clearly defined outcome measures, and transparent statistical analyses. The three case–control studies had high methodological quality according to the Newcastle-Ottawa Scale, which reflected appropriate case selection, comparability between groups, and robust exposure assessment.

### 4.1 Strengths

Adherence to the Joanna Briggs Institute method and reporting as per the PRISMA-ScR extension is one strength of this scoping review. The use of modern molecular and genomic techniques along with *Prakṛti* analysis in the studies supports future mechanistic investigation because of the attempt to move beyond analysis only by *Āyurvedic* conceptual parameters and to bring the studies on a more molecular correlative footing (33–48). The use of large, robust sample sizes in eight of the studies (34, 36, 38, 40, 42, 44, 46, 47) may improve statistical power, subgroup analysis, potential for reproduction, and confidence in associations. Another key strength is deep phenotyping, which is the systemic characterization of an individual’s *Prakṛti* integrated with multidimensional biological data. Techniques of deep phenotyping across the studies included standardized *Prakṛti* assessment; phenotyping based on clinical, physiological, and biochemical parameters; genetic and genomic profiling; and systems biology approaches. These techniques improve internal validity by reducing overlap between categories of *Prakṛti* and enabling easier detection of genomic differences. The studies assessing the gut and oral microbiomes (42, 46), olfactory receptor function gene expression with perception of smell (36), and differences in cell proliferation in lymphoblastoid cell lines (41) assess multidomain correlation, which reflects biological variability. The use of the STREGA checklist and established frameworks for quality appraisal and reporting (AXIS tool and Newcastle-Ottawa Scale) ensures transparency, systemic evaluation of bias, and adherence to genetic epidemiology reporting standards (33–48).

### 4.2 Limitations

The lack of standardization of assessing *Prakṛti* makes the studies vulnerable to factors such as subjectivity, inter-variability, and reproducibility, and thus presents a major concern in the robust and smooth execution of studies in *Āyurgenomics*. Correlation does not equal causality in these studies as they are observational, cross-sectional, and association-based; hence, exact causal relationships in biological pathways cannot be established, and many findings themselves are only exploratory. Replication is limited with these studies because of their absence across multiple populations, with some studies being population-specific. Small cohorts seen in eight studies (33, 35, 37, 39, 41, 43, 45, 48) make the findings hypothesis-generating as opposed to definitive. In addition, these may increase the risk of bias in population stratification, false positives, and population over-representation. None of the studies conclusively demonstrate the prospective prediction of *Prakṛti* to disease, the impact of *Prakṛti-*guided therapeutic outcomes, and the stability of genomic outcomes. Exact correlations between *Prakṛti* and disease cannot be determined due to probabilistic associations; the studies only suggest tendencies or enrichments, not deterministic mappings. Inconsistencies in the reporting of parameters such as sensitivity analyses, participant flow, handling of missing data, and potential sources of bias were observed across the studies according to the STREGA checklist. Information about non-responders was generally not applicable or was not reported due to the selection of subjects from predefined cohorts or convenience samples. Consequently, assessment of non-responder bias could not be adequately assessed, which limited the evaluation of representation and generalization of some of the findings. Although the AXIS tool was applied systematically, adequate discriminatory rigor was limited across the studies. Effective differentiation according to overall methodological quality or risk of bias could not be done, despite identifying methodological strengths and weaknesses. This may be due to the homogeneous nature of the study methodologies and that the primary aim of the AXIS checklist is to report methodological elements and not to grade their overall quality.

### 4.3 Role of *Āyurgenomics* in primary healthcare w.s.r. to the included studies

The role that *Āyurgenomics* can play in the primary healthcare sector may be understood under several broad heads: early risk identification and preventive healthcare, personalized dietary and lifestyle counseling, strengthening precision public health, low-cost community-based risk stratification, integration of traditional knowledge with modern medicine, and role in managing chronic disease (52–59). The expansion of each aspect and its explanation based upon the selected studies of this scoping review is provided in Table 8.

**Table 8:** Role of selected studies in primary healthcare.

Aspects of primary healthcare	Example of study	Expansion
Early-Risk identification and preventive healthcare	Murugan et al., 2025 (38)	*Prakṛti-*based stratification can identify individuals predisposed to chronic, non-communicable disease. They can receive various interventions through a PHC approach (52). Early identification may reduce the risk of metabolic and other disorders (53).
	Singh et al., 2024 (33)	
	Abbas et al., 2022 (39)	
	Gupta et al., 2018 (35)	
	Rotti et al., 2015 (48)	
Personalized dietary and lifestyle counseling	Chakraborty et al., 2021 (41)	Individuals respond differently to dietary, environmental, and lifestyle stressors. Personalized counseling on aspects such as diet, lifestyle, sleep, exercise, and preventive regimens may improve patient engagement and lead to better adherence and clinical outcomes (54, 55).
	Chauhan et al., 2018 (42)	
	Prasher et al., 2008 (44)	
	Shalini et al., 2021 (46)	
Strengthening precision public health	Juyal et al., 2022 (40)	Population subgroups possess distinct biological and genetic characteristics. Identification of vulnerable groups and tailoring of precision programs by leveraging a variety of data and utilizing emerging technology methods to improve public health and reduce disparities (56).
	Aggarwal et al., 2015 (43)	
	Aggarwal et al., 2010 (45)	
	Govindaraj et al., 2015 (47)	
Low-cost community-based risk stratification	Studies employing questionnaire-based *Prakṛti* assessment proformas (33–35, 38, 40, 42–44)	This may be applied in resource-limited settings, given the increasing cost and utilization of healthcare services (57). Community healthcare workers may utilize them for risk stratification prior to laboratory investigations.
Integration of traditional medicine with modern healthcare	Juyal et al., 2022 (40)	Biological correlations between *Āyurvedic* constitutional types and variations between genome, metabolome, microbiome, and phenotype. Traditional medicine can enhance PHC delivery, especially in underserved settings (58). Integrative healthcare models incorporating Ayurveda and biomedicine can be encouraged due to AYUSH playing a pivotal role in achieving Global Health Coverage (58).
	Chauhan et al., 2018 (42)	
	Prasher et al., 2008 (44)	
Potential role in managing chronic disease	Trivedi et al., 2026 (37)	*Prakṛti-*based approaches may help predict disease severity and progression. Integrated care systems are a proposed model for managing chronic disease that collaborate by bringing together various systems of healthcare. This can aid in improving population healthcare outcomes and reduce duplication and inefficiency in emerging nations (59).
	Juyal et al., 2012 (34)	

### 4.4 Challenges and future scope of *Āyurgenomics* research

Although *Āyurgenomics* holds significant potential, there exist challenges to implementing it, especially in the primary healthcare sector. The first major step toward broader acceptance is to address these. Extensive, robust, and rigorous scientific evaluation and validation of fundamental principles, incorporating multi-centric studies, is essential. These initiatives may be successful with interdisciplinary collaborative studies involving practitioners of traditional, complementary, and alternative medicine (TCAM), and modern scientists and physicians (60). Improvements in reporting transparency and assessment of bias can further strengthen the scope of research in *Āyurgenomics*, especially in the interpretation of evidence. Quality appraisal should be descriptively analyzed and interpreted rather than incorporated as a means to rank the evidence. Other potential areas of research in light of the selected studies can include artificial intelligence (AI)-based *Prakṛti* assessment tools, genome-wide association studies (GWAS) in *Āyurveda,* interactions between *Prakṛti* and the microbiome, precision public health models, and identification of *Āyurvedic* biomarkers for chronic illnesses. Research on the effect of *Āyurgenomics* in different types of meditation, including Maharishi Mahesh Yogi’s Transcendental Meditation, may also be conducted in this regard. In addition to these, standardized *Prakṛti* assessment, longitudinal validation, replications across various populations, functional mechanistic studies, and clinical outcome trials are imperative. While conducting these, clear guidelines for the ethical use of *Āyurgenomics* and navigating intellectual property rights related to traditional knowledge are imperative (60, 61).

## 5 Conclusion

Current *Āyurgenomics* literature supports the idea that *Prakṛti* may correspond to measurable biological variation. However, the present evidence is not robust enough to claim exact deterministic correlations with disease. This scoping review collates the studies on *Āyurgenomics* with special reference to studies incorporating both analysis of *Prakṛti* and genetic assessment. Conclusions drawn from each study reflect the potential of *Āyurgenomics* to be applied in the respective condition of assessment. The field shows scientific promise but requires further robust studies. These may be executed to add to and enhance already-existing knowledge about *Prakṛti* and its association with the genomic template of an individual.

## Data Availability

The original contributions presented in the study are included in the article/supplementary material, further inquiries can be directed to the corresponding author/s.
